# Variability in protocols on donation after circulatory death in Europe

**DOI:** 10.1186/cc13034

**Published:** 2013-10-03

**Authors:** Jentina Wind, Marloes Faut, Tim C van Smaalen, Ernest LW van Heurn

**Affiliations:** 1 Department of Surgery, Maastricht University Medical Centre, P.O. Box 5800, 6202 AZ Maastricht, The Netherlands

## Abstract

**Introduction:**

Organ donation after circulatory death (DCD) has become an accepted strategy to reduce the shortage of organs for transplantation in many European countries. The use and number of DCD donors varies between countries. The purpose of this study was to evaluate the available protocols for DCD in Europe.

**Methods:**

We contacted national transplant societies and responsible transplant co-ordinators in the countries that perform DCD to obtain DCD protocols. We compared information on the protocols and additional data including: inclusion and exclusion criteria for donation, legislation, determination of death and preservation methods.

**Results:**

In ten European countries DCD is performed, eight of which describe the methods in protocols. There are large differences in used DCD categories, legislation and the way death is determined. Protocols differ in the detail in which DCD procedures are described and the way methods are supported by additional consensus statements and ethical frameworks.

**Conclusions:**

Although DCD is an established strategy to enlarge the donor pool and to contribute to the reduction of the waiting list for transplantation, its potential has not been fully utilized yet. To further promote DCD transplantation, it is important to share expertise and obtain consensus, so that this can be translated into more uniform and solid protocols supported by the competent authorities, transplant and intensive care professionals, which may eventually result in a further promotion of DCD transplantation in Europe.

## Introduction

Kidney transplantation increases life expectancy and quality of life in patients with end-stage renal disease compared with renal dialysis [1,2]. However, there is an ongoing universal shortage of donor organs to meet the demand for transplantation, and organ donation and transplantation rates vary widely across countries. The World Health Organization published guiding principles on human cell, tissue and organ transplantation to support countries in taking responsibility for progression to self-sufficiency for the organ donation and transplantation needs of their people [3].

One way to expand the organ donor pool is the use of organs from donors who die after circulatory death (DCD). These organs are inevitably subjected to a period of warm ischemia, which adversely affects transplant outcome [4]. Despite the higher incidence of primary nonfunction and delayed graft function, transplantation of DCD kidneys is associated with increased recipient survival compared with staying on the transplant waiting list with the option of later receiving a conventional kidney from donation after brain death (DBD) [5]. Procurement of kidneys from DCD donors holds the potential to expand the donor pool 2.5 to 4 times [6].

Next to DCD kidney transplantation, which has been a generally accepted source of donor organs in many countries, DCD transplantation of the liver, lung and pancreas is possible and has been increasingly performed in the past decade. The results of DCD liver transplantation are comparable with DBD liver transplantation, with equivalent 1-year and 3-year graft survival and patient survival, although there is a higher risk of biliary strictures in DCD livers [7]. The number of DCD lung transplantations is still relatively low; a number of transplant centers show equivalent outcomes for DCD and DBD lung transplantation. DCD lung transplantation offers a good opportunity to reduce the donor lung shortage [8]. The DCD potential is still not fully used, despite the fact that DCD has proven to be effective to expand the donor pool. In contrast to DBD, DCD is not performed in every European country and the percentage of DCD donors out of the total number of deceased donors varies largely.

In 1995, at the First International Workshop on Non-heart Beating Donation in Maastricht, four categories of nonheart-beating donation were identified, which include controlled and uncontrolled donors (Table 1) [9]. Nomenclature has changed since then, from nonheart-beating donation to donation after cardiac death, to make a more clear difference between cardiac death and brain death. Recently, donation after circulatory death has been proposed as the nomenclature of choice.

**Table 1:** Maastricht classification of donors after circulatory death

**Category**	**Description**	**Procurement**
I	Dead on arrival	Uncontrolled
II	Unsuccessful resuscitation	Uncontrolled
III	Awaiting cardiac arrest	Controlled
IV	Cardiac arrest while brain dead	Uncontrolled

Depending on the category, the donor procedure is carried out in the emergency room, in the ICU or in the operating room. The popularization of DCD has caused a growing demand for protocols that describe donor management and preservation strategies. Furthermore, ethical aspects of DCD are essential and have to be addressed [10,11].

The aim of this study is to evaluate European DCD protocols, focusing on the inclusion and exclusion criteria for organ donation, legislation on DCD donation, determination of death and preservation methods for organ procurement. Although protocols do not always completely reflect the actual clinical practice and often small violations of the protocols are present, this evaluation may provide a general overview of the European attitude towards DCD. It is important to obtain an overall understanding of the different approaches to DCD in Europe and to evaluate whether there is room for improvement.

## Materials and methods

There are currently 10 countries in Europe with an active DCD program [12]. Between January 2011 and January 2012 we directly contacted national transplant societies in these countries and responsible transplant coordinators, and obtained their national and regional DCD protocols (Table 2). If the information was not complete, we were referred by the transplant societies to specific transplant centers and responsible representatives. Information of recent publications, the database from the Transplant Procurement Management organization and published national consensus meetings was used to further complete the information. From these data, we compared the national strategies in DCD donation, including inclusion and exclusion criteria for DCD donation, legislation, determination of death and preservation methods. The study was performed in agreement with the code of conduct on the use of data in health research, put forward by the Dutch Federation of Biomedical Scientific Societies [13].

**Table 2:** National authorities for organ donation and responsible organizations for organ allocation in different countries

**Country**	**National authority for organ donation**	**Responsible organization for organ allocation**
Austria	Österreichisches Bundesinstitut für Gesundheitswesen (ÖBIG)	Eurotransplant
	[http://www.goeg.at]	
Belgium	Belgische Transplantatie Vereniging/Société Belge de Transplantation/Belgian Transplant Society (BTS)	Eurotransplant
	[http://www.transplant.be]	
Czech Republic	Koordinační Středisko Transplantací (KST)	Koordinační Středisko Transplantací
	[http://www.kst.cz]	
France	Agence de la Biomédecine	Agence de la Biomédecine
	[http://www.agence-biomedecine.fr]	
Italy	Centro Nazionale Trapianti (CNT)	Centro Nazionale Trapianti
	[http://www.trapianti.salute.gov.it]	
Latvia	BaltTransplant – Latvian subdivision	BaltTransplant
	[http://www.stradini.lv]	
The Netherlands	Nederlandse Transplantatie Stichting (NTS)	Eurotransplant
	[http://www.transplantatiestichting.nl]	
United Kingdom	National Health Service Blood and Transplant (NHSBT)	National Health Service Blood and Transplant
	[http://www.nhsbt.nhs.uk]	
Spain	Organización National de Trasplantes (ONT)	Organización National de Trasplantes
	[http://www.ont.es]	
Switzerland	Swiss Transplant	Swiss Transplant
	[http://www.swisstransplant.org]	

## Results

Ten countries in Europe actively perform DCD, of which eight have specific protocols for DCD or describe DCD procedures in one general protocol for organ donation. Latvia and Czech Republic have no specific description of DCD donation mentioned in their protocol. In four countries (Italy, Austria, Latvia, Czech Republic), DCD activity is restricted to one or two hospitals. In the Netherlands and the UK, DCD donation is possible in almost every hospital. Ten other countries are planning to start a DCD program in the near future, including Cyprus, Estonia, Luxembourg, Norway, Poland, Portugal, Romania, Slovak Republic, Slovenia and Sweden. In seven countries (Finland, Germany, Greece, Bosnia-Herzegovina, Hungary, Lithuania, Turkey) there is no planned DCD activity, mainly because of legal restriction. The most important reason for not performing DCD next to legislation includes organizational difficulties [12].

Table 3 summarizes the donor registration system, the DCD categories used for donation and the organs procured from DCD donors in different European countries. Reasons to explain the absence of controlled DCD donation (Maastricht category III) were ethical and juridical restraints (France, Spain, Latvia); uncontrolled DCD (Maastricht category I and II) was often not performed because of organizational difficulties.

**Table 3:** Registration system, donation after circulatory death categories, and retrieved organs in different countries

**Country**	**Registration system**	**DCD categories**	**Organs retrieved**
Austria	Opt out	II, III	Kidneys
Belgium	Opt out	II, III, IV^a^	Kidney, liver, pancreas, lungs^a^
Czech Republic	Opt out	I,II, III, IV	Kidneys
France	Opt out	I, II, IV	Kidneys
Italy	Opt out	II, III, IV	Kidneys
Latvia	Opt out	II, IV	Kidneys
The Netherlands	Opt in	II, III, IV	Kidneys, liver, pancreas, lungs
United Kingdom	Opt in	II, III, IV	Kidneys, liver, pancreas, lungs^a^
Spain	Opt out	I,II,IV^a^	Kidney, liver, lungs
Switzerland	Opt in	I,II,III,IV	Kidney, liver, lungs

DCD, donation after circulatory death. ^a^Variation in regional protocols.

### Inclusion and exclusion criteria

Out of the 10 countries with DCD, seven countries (70%) described inclusion and exclusion criteria of controlled and/or uncontrolled donation. Seven countries actively performed controlled as well as uncontrolled DCD (Spain, France and Latvia only uncontrolled DCD), of which four separated inclusion and exclusion criteria for controlled and uncontrolled donation in their protocols, which mainly included differences in donor age limits. Inclusion criteria for donation generally included positive registration as a donor or permission of the next of kin if a donor had not been registered with a donor registry, witnessed arrest (in uncontrolled DCD) and age limits. The most commonly used exclusion criteria were hypothermia, intoxication, malignancy, sepsis (untreated) and organ-specific contra-indications such as renal failure and liver cirrhosis. Next to the internationally used Maastricht DCD categories, Spain and Italy describe additional categories that include donation after sudden irreversible cardiac arrest in the ICU (Spain) and death during extracorporeal membrane oxygenation (Italy). In most countries DCD donors are predominantly Maastricht category III donors – that is, donors who die after withdrawal of treatment – which results in a high percentage of DCD donors in the UK, the Netherlands and Belgium (Table 4). France performs only uncontrolled DCD. Spain recently performed its first controlled DCD, after many years of successful uncontrolled DCD.

**Table 4:** Absolute numbers and percentage of DCD donors in 2011 and 2012 in different countries

**Country**	**Actual DCD donors**		**Percentage of DCD donors from the total deceased donors**		**Number of deceased donors (PMP)**		**DCD since (year)**
	**2011**	**2012**	**2011**	**2012**	**2011**	**2012**	
Austria	6	4	3	2	23.2	22.5	1994
Belgium	61	70	19	19	29.3	32.9	1994
Czech Republic	1	2	<1	1	17.6	19.8	2002
France	58	n/a	4	n/a	25.0	n/a	2006
Italy	6	5	<1	1	21.9	22.4	2005
Latvia	13	23	32	60	17.9	19.0	1992
The Netherlands	111	124	50	49	13.2	15.0	1981
United Kingdom	405	n/a	38	n/a	17.0	n/a	1989
Spain	117	161	7	10	35.3	34.8	1989
Switzerland	3	7	3	7	12.8	12.0	1993

Absolute numbers and percentage of DCD donors of the total number of deceased donors in 2011 and 2012 in different countries. DCD, donation after circulatory death; PMP, percentage per million population.

### Legislation

In all countries with a donor protocol, there was an organ donation law. Although the actual donation act was often not included in the protocol, a reference was always available. Three out of 10 countries (30%) performing DCD in Europe have an opt-in registration system; this means that explicit consent for donation is necessary by donor card or registration in a national registry (Table 3). In the other seven countries (70%) with a DCD program, presumed consent is used; explicit consent for donation is not required but is presumed if the person has not objected during life.

### Opt-in registration

There are three countries with a DCD program and an opt-in registration system (the UK, the Netherlands and Switzerland). In the Netherlands and Switzerland, registration as a donor is sufficient to proceed to organ procurement, and the relatives are informed. In the UK, the opt-in registration system requires additional consent by the next of kin. A registered objection to donation always excludes organ donation.

All three protocols allow use of *in situ* preservation (ISP) in uncontrolled DCD donors to preserve organs if a person has not objected to donation. ISP is a minimally invasive technique to preserve the organs after determination of death and a no-touch period. A special catheter with proximal and distal occlusion balloons is inserted through the femoral artery into the aorta, which enables selective flushing of the kidneys. ISP preserves organ viability and provides the opportunity to meet legal and logistical requirements before organ procurement. ISP also gives relatives time to decide about organ donation.

### Opt-out registration

Seven countries use the system of presumed consent for donation. Six out of seven protocols require additional permission from the next of kin; Spain requires written permission. Austria does not specify informed consent of the relatives in its protocol. In clinical practice, in most countries with an opt-out system, the relatives are consulted to confirm the consent for donation. In Latvia, the next of kin has to submit their objection in writing if there is no information in the donor registry. In uncontrolled donors, most protocols allow ISP awaiting the relatives’ consent. In Spain, approval has to be requested from the local court. In most countries, it is allowed or otherwise not specifically mentioned in the protocol for controlled donors, to maintain or optimise the condition of the potential donor after the decision has been made to withdraw treatment if this does not harm the patient or cause discomfort. In Switzerland, (opt-in) consent of the next of kin is required before starting any medical treatment to improve the condition of the donor.

### Determination of death

The description of how death is determined varied in the protocols; from no description at all, or referring to standard criteria (the so-called *lege artis*), to more specific guidelines and tests that have to be performed (Table 5). Some protocols refer to guidelines or protocols for the determination of death [14,15]. The standard criteria to determine death after cessation of medical treatment of a DCD donor refer to cardiocirculatory criteria: irreversible cessation of circulatory and respiratory function. Diagnostic procedures used to confirm death differ among protocols; absent intra-arterial pressure, isoelectric electrocardiogram, absent cardiac function on echocardiogram (Table 4). All protocols describe an observation period after circulatory arrest – the no-touch time, the period of time during which no interventions are performed in the donor. However, this period is described differently: as the observation period between the circulatory arrest and the determination of death, or as the time period between determination of death and initiation of organ procurement. The no-touch time differs from 5 to 20 minutes (Table 5). In Italy, 20 minutes of an isoelectric electrocardiogram registration is necessary very difficult.

**Table 5:** Items described in the protocols

**Country**	**Criteria for determination of death and diagnostic procedures**	**Death determined by**	**No-touch time (minutes)**	**Preservation method**
Austria	Asystole, not specified	Treating physician with *jus practicandi*	10	Laparotomy with direct cannulation
Belgium	Cardiorespiratory criteria, according to most recent standard^a^	Three independent physicians	5	Laparotomy with direct cannulation
Czech Republic^b^	Not described	Independent physician, not involved in donation	10	DBTL catheter
France	Cardiorespiratory criteria, unconsciousness, absence of brainstem reflexes. ECG	Independent physician, not involved in donation	5	nECMO, DBTL catheter
Italy	Asystole, isoelectric ECG to confirm	Treating physician	20	nECMO
Latvia^b^	Not described	Intensivist	15	Laparotomy with direct cannulation, DBTL catheter
The Netherlands	Cardiocirculatory arrest, not specified	Treating physician	5	Laparotomy with direct cannulation, DBTL catheter
United Kingdom^a^	Cardiocirculatory arrest, unconsciousness. Intra-arterial pressure monitoring, ECG during 5 minutes. After 5 minutes, absence of brainstem reflexes confirmed	Treating physician	5	Laparotomy with direct cannulation, DBTL catheter
Spain	Asystole, apnea, no response to stimuli. ECG to confirm	Treating physician	5	ECMO, nECMO, DBTL catheter
Switzerland	*Lege artis*, referred to specific guidelines, TTE to confirm asystole	Two independent physicians	10	Laparotomy with direct cannulation

DBTL, double balloon, triple lumen; ECG, electrocardiogram; ECMO, extracorporeal membrane oxygenation; nECMO, normothermic extracorporeal membrane oxygenation; TTE, transthoracic echocardiogram.

^a^Variation in regional protocols. ^b^Donation after circulatory death not described in a protocol.

Separation of the team responsible for the treatment of the patient and the organ procurement team is mentioned in all protocols. In Spain and France, the potential uncontrolled donor is recognized by the ambulance service and, after unsuccessful resuscitation, treated as a potential donor with continuation of chest compressions, ventilation and fluid perfusion. Only after arrival at the hospital does the attending physician determine death of the patient and the procurement team starts the donor procedure.

### Preservation methods

The most commonly used preservation method for controlled DCD donation is rapid laparotomy with cannulation of the abdominal aorta [16]. After death in the ICU, the deceased is taken to the operating room. In uncontrolled donors, ISP with the double-balloon, triple-lumen catheter or the Gillot catheter is used. In the two countries with the largest numbers of uncontrolled donors (Spain and France), extracorporeal membrane oxygenation with either hypothermic or normothermic perfusion is used as the preservation method of choice.

## Discussion

There is a relatively large variability in European DCD protocols. This can be expected, because legislation, expertise, experience and organizational factors differ. Especially uncontrolled DCD requires specific organization, because the donation procedure is always unexpected. The time between the determination of death and preservation of the organs is important, to minimize warm ischemic time and organ injury. The way in which DCD procedures are described varies, from no specific protocol (two countries) to detailed protocols with complementary consensus statements and ethical frameworks, as in the UK [17].

Reasons for not performing DCD at all include conflicts with national law or difficulties with the infrastructure to initiate a DCD program [12]. Also, reluctance to explore the possibilities within the law to perform DCD can play a role. Countries that perform DCD differ in the percentage of DCD transplantations compared with the total numbers of deceased donors, and the use of different Maastricht categories. Some countries focus on uncontrolled DCD (Spain, France), and others focus on controlled DCD (the Netherlands, the UK) or perform both. The difference can be explained by practice in ICUs to withdraw life-sustaining treatment or not. In northern European countries, withdrawal of treatment, if treatment is considered futile, is a more accepted practice than in southern European countries [18]. These differences may partly be caused by the availability of intensive care capacity, which may force doctors towards less expectative approach to discontinue useless medical treatment.

A notable difference between protocols is the way in which death of the patient is determined. When a deceased patient is an eligible organ donor, both the accurate determination of death and a fast transition from treating a patient to preserving a donor is essential to enable organ preservation with minimized ischemic injury to the organs. Definitions of the criteria to determine death and the no-touch period, to ensure irreversibility of cardiocirculatory arrest, are therefore crucial. Determination of death varies in the different protocols, but also between ICUs. There is a lack of uniformity [19-21]. This includes the definition of death, the tests used to determine death and the period of time between death and the moment organs can be procured. Different definitions have important implications for donor treatment and end-of-life care, but also for the ischemic period of the organs and organ viability. With the expansion of DCD programs, the lack of consensus about determination of death is a point of discussion and concern [22-24]. It is important to have clear, uniform and consistent evidence-based guidelines to determine death, in order to fulfill medical, ethical and legal obligations and to ensure public trust [19].

Donor procurement strategies differ throughout Europe, and the implementation of protocols differs even more. Strategies to procure organs each have their advantages and disadvantages and depend on consent systems, legal opportunities, in-hospital and out-of-hospital institution and financial possibilities. Protocols can be helpful to obtain and maintain support for DCD programs and to help other institutes or countries to develop, implement and continue DCD. The protocols also provide answers to important ethical questions concerning end-of-life management and care [25].

## Conclusion

DCD is an established strategy to enlarge the donor pool and to contribute to the reduction of the waiting list for transplantation. The DCD potential in Europe is not yet fully utilized. DCD protocols in European countries and even in individual hospitals are heterogeneous. Restrictions by national laws, organizational problems or ethical struggles are important factors for this. Following these conclusions, we feel it is important that experiences with DCD are shared and consensus is obtained, so these can be translated into more uniform and solid protocols supported by the competent authorities, transplant and intensive care professionals, which may eventually result in a further promotion of DCD transplantation in Europe.

## Key messages

• Transplantation of organs from donors after circulatory death (DCD) contributes to the reduction of the waiting list for transplantation, but the DCD potential in Europe is not yet fully utilized.

• There is a large variability within European countries to perform DCD and existing DCD protocols are heterogeneous.

• Protocols can be helpful to develop, implement and improve DCD performance and answer ethical questions.

## Abbreviations

DBD: Donation after brain death; DCD: Donation after circulatory death; ISP: *In situ* preservation.

## Competing interests

The authors declare that they have no competing interests.

## Authors’ contributions

JW conceived and designed the study, analyzed the data and wrote the manuscript. MF collected the data and revised the manuscript. TCvS collected the data and revised the manuscript. ELWvH designed the study and helped to draft the manuscript. All authors read and approved the final manuscript for publication.
